# Aberrant expression of ZNF268 alters the growth and migration of ovarian cancer cells

**DOI:** 10.3892/ol.2013.1318

**Published:** 2013-04-24

**Authors:** LI HU, WEI WANG, JINYANG CAI, JUN LUO, YI HUANG, SHILU XIONG, WENXIN LI, MINGXIONG GUO

**Affiliations:** 1 State Key Laboratory of Virology, College of Life Sciences, Wuhan University, Wuhan, Hubei 430072;; 2 Department of Pathology, Zhongnan Hospital, Wuhan University, Wuhan, Hubei 430071;; 3 Department of Gynecologic Oncology, Hubei Cancer Hospital, Wuhan, Hubei 430079, P.R. China

**Keywords:** ZNF268, ovarian cancer, SKOV-3 cells

## Abstract

Ovarian cancer is one of the most lethal gynaecological cancers worldwide. However, the mechanisms underlying ovarian carcinogenesis are not well understood. The present study used immunostaining, western blotting and quantitative real-time PCR to demonstrate that ZNF268 is overexpressed in human ovarian carcinomas. ZNF268-knockdown increased the viability, colony formation and growth of *in vivo* xenografts of ovarian carcinoma SKOV-3 cells, whereas SKOV-3 cell migration was inhibited. Furthermore, it was demonstrated that the knockdown of ZNF268 may increase SKOV-3 cell growth by promoting cell cycle progression. The findings suggest that ZNF268 is a novel protein involved in ovarian carcinogenesis and that it may aid in the understanding of the mechanisms of ovarian carcinogenesis.

## Introduction

Krüppel-associated box (KRAB)-containing zinc finger (KRAB-ZNF) proteins, which represent the largest single family of transcriptional regulators in mammals (1), have been shown to regulate gene expression by binding to target DNA sequences through the zinc finger domain, thereby allowing KRAB to repress transcription (2,3). However, little is known concerning the biological functions of KRAB-ZNF proteins (4). ZNF268, which was isolated from a human embryo cDNA library (5), is a typical KRAB-containing zinc finger protein that has been observed to produce eight splice variants and is translated into two proteins, ZNF268a and ZNF268b2 (6). ZNF268a contains a KRAB domain and up to 24 zinc fingers and may function as a transcriptional repressor (7), while ZNF268b2 consists of only the zinc finger domain and contributes to human cervical cancer via the NF-κB signalling pathway (8). The ZNF268 promoter is located in the first exon of the gene and is regulated by cAMP response element binding protein 2 (CREB-2) (9). Previous studies have suggested that ZNF268 may be involved in human foetal liver development (10), haematological diseases (11–13) and cervical cancer development (8). Based on tissue microarray results, ZNF268 may be a multifunctional molecule that functions as either a promoter or a suppressor, depending on the cancer subtype (8). This hypothesis is supported by findings indicating that ZNF268-knockdown promotes the proliferation of erythroleukemia K562 cells (14), while inhibiting the growth of cervical cancer HeLa cells (8). However, the function of ZNF268 in ovarian tissues remains to be determined.

Ovarian cancer is one of the most lethal types of gynaecological cancer and the seventh leading cause of cancer mortality among females worldwide (15). Its incidence in Asian countries is increasing (16). The high mortality associated with ovarian cancer is largely due to the asymptomatic nature of early stages of the disease (prior to the development of widespread metastases) and the significant failure rate of chemotherapy for curing the advanced disease (17,18). At present, the pathogenic mechanisms underlying the development of human ovarian cancers are too complicated to be fully understood (19). Therefore, there is an urgent requirement to investigate the cellular and molecular mechanisms underlying ovarian carcinogenesis and identify novel therapeutic targets that improve the survival of patients with ovarian cancer.

The present study demonstrated that ZNF268 was overexpressed in human ovarian cancer tissues and that ZNF268-knockdown increased the proliferation, while simultaneously decreasing the migration, of SKOV-3 ovarian cancer cells. The effects of ZNF268 on SKOV-3 cell growth may be mediated by altering cell cycle progression. The results obtained from the current study are likely to provide an improved understanding of the molecular mechanisms underlying ovarian cancer progression and demonstrate the potential of ZNF268 as a novel therapeutic target for ovarian cancer.

## Materials and methods

### Cell culture, tissue specimens and immunohistochemistry

SKOV-3 cells (CCTCC, Wuhan, China) were grown in McCoy’s 5A medium supplemented with 10% fetal bovine serum (FBS; Invitrogen, Carlsbad, CA, USA), penicillin (100 units/ml) and streptomycin (100 *μ*g/ml) at 37°C in a 5% CO_2_ incubator. Four paraffin-embedded normal ovarian specimens and 20 paraffin-embedded ovarian carcinoma specimens were obtained during routine clinical practice from female patients who were undergoing either biopsy or surgery at the Department of Pathology, Zhongnan Hospital, Wuhan University (Wuhan, Hebei, China). The study was approved by the Regional Committee of Medical Research Ethics in China and informed consent was obtained from each patient.

All immunohistochemical staining assays were performed by the Jiayuan Quantum Dots Company (Wuhan, China) according to the standard procedure. The expression levels of the examined proteins were scored by two independent pathologists who had no knowledge of the clinical or histopathological data. The expression levels were scored as follows: 0 points (−, no detectable staining), one point (+, weak staining), two points (++, clear but not strong staining) and three points (+++, marked staining) (20).

### Establishment of ZNF268 stable knockdown SKOV-3 cells

SKOV-3 cells were seeded in 60-mm dishes at a density of 3.0×10^4^ cells/dish. When the cells had reached ∼70% confluence, they were infected with shZNF268 or sh control lentiviral particles as described previously (14). Flow cytometry was used to sort the GFP-positive cells. Reduced ZNF268 expression was confirmed at the mRNA and protein levels (14).

### Western blotting

The cells were collected and lysed in RIPA buffer on ice for 15 min, followed by centrifugation at 16,000 x g at 4°C for 10 min. The supernatants were collected and subjected to western blotting according to the standard procedure as described previously (8). The antibodies used for the western blotting analysis were as follows: Actin antibody was purchased from Santa Cruz Biotechnology, Inc. (Santa Cruz, CA, USA); cyclin D2, cyclin E2 and CDK2 antibodies were purchased from Cell Signaling Technology (Danvers, MA, USA); and anti-SD antibody for the detection of ZNF268 (produced in our lab) was used as described previously (6).

### RNA isolation, reverse transcription and quantitative real-time PCR

RNA was extracted using TRIzol reagent (Invitrogen). cDNA was prepared according to the manufacturer’s instructions (Toyobo, Osaka, Japan). Real-time PCR was performed and analysed using an ABI 7500 detection system. The relative mRNA levels of ZNF268 in each sample were normalised to GAPDH as described previously (14).

### MTT assay

The SKOV-3 cells were plated on 96-well cell culture plates at a density of 2.0×10^3^ cells/well. At designated time-points, the cells were incubated with 20 *μ*l 3-(4,5-dimethylthiazol-2-yl)-2,5-diphenyltetrazolium bromide dye (MTT, 5 mg/ml) for 2 h, followed by solubilisation for 10 min in DMSO (100 *μ*l/well), with agitation at room temperature. The absorbance was determined at 570 nm using a microplate reader (El×800, BioTek Instruments, Inc., Winooski, VT, USA).

### Soft agar assay

The cells were trypsinised and suspended in 2 ml top agar containing 10% FBS and 0.3% agarose. The mixture was then plated onto 60-mm dishes containing 2 ml bottom agar with 10% FBS and 0.6% agarose. Subsequent to incubation for 3 weeks, 1 ml of a 1 mg/ml solution of 2-(p-iodophenyl)-3-(p-nitrophenyl)-5-phenyl tetrazolium chloride was added. After 4 h, images of the plates were captured and the number of colonies was counted.

### Cell cycle progression and apoptosis assays

Cell cycle progression and apoptosis were assessed using flow cytometric analysis to measure the DNA content. Briefly, the SKOV-3 cells (1.0×10^6^) were collected, washed twice with PBS and resuspended in ice cold 70% ethanol for 2 h at 4°C. The cells were then washed twice with PBS and digested using RNase A (1 mg/ml) at 37°C for 30 min. The cells were stained with propidium iodide (PI, 5 *μ*g/ml) for 1 h at 4°C and analysed using a Beckman Coulter Epics XL flow cytometer (Beckman Coulter, Miami, FL, USA).

### Nude mouse tumour formation assay

Five-week-old male nude mice (Balb/c nu/nu) were purchased from SJA Lab Animal Limited Company (Changsha, China). The animals were housed in a specific pathogen-free facility and maintained in a temperature-controlled environment on a 12-h light/dark cycle with free access to sterilised food and autoclaved water. The SKOV-3 cells in the exponential growth phase were trypsinised into single-cell suspensions and injected subcutaneously into the nude mice. All mice were maintained for 30 days prior to being sacrificed. All animal experiments were approved by the Animal Research Ethics Board of Wuhan University and were conducted in compliance with the institutional guidelines for the care of experimental animals.

### Wound healing migration assay

Either the SKOV-3 or HeLa cells (1×10^5^) were plated onto six-well plates and allowed to form a confluent monolayer. The cell monolayer was then scratched in a straight line to make a ‘scratch wound’ with a 0.2-ml pipette tip and the cell debris was removed by washing the cells with phosphate-buffered saline. McCoy’s 5A medium (for the SKOV-3 cells) or DMEM medium (for the HeLa cells) supplemented with 1% FBS was added, and images of the closure of the scratch were captured at 0, 24 and 48 h.

### Statistical analysis

The data are represented as the mean ± SD of the samples. All experiments were repeated three times. A two-tailed Student’s t-test was used to compare the differences between the two experimental groups. P<0.05 was considered to indicate a statistically significant difference.

## Results

### ZNF268 is overexpressed in human ovarian cancer tissues

**Figure 1.**
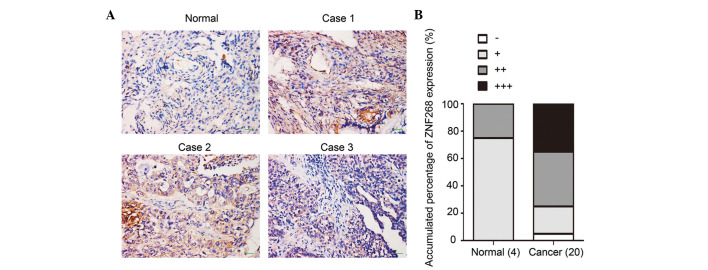
ZNF268 expression in human ovarian tissues. (A) Representative images of ZNF268 immunohistochemical staining in ovarian tissues. Three cases of cancerous ovarian tissues are shown. Anti-SD antibodies were used to detect ZNF268 expression using immunohistochemistry. The scale bars represent 50 *μ*m; magnification, ×400. (B) Accumulated percentage of ZNF268 expression in normal and cancerous ovarian tissues. The numbers in brackets indicate the number of specimens investigated.

We previously observed different expression patterns of ZNF268 in normal human ovarian tissues compared with ovarian cancer tissues using the tissue microarray method. ZNF268 was overexpressed in the majority of the ovarian cancer tissues (∼84%), while ZNF268 expression was rarely detected in the normal tissues (8). To confirm these results, more ovarian tissues (four normal and 20 cancerous specimens) were obtained and subjected to immunohistochemistry with an anti-SD antibody to detect ZNF268 expression (6). The majority of the cancerous samples exhibited high levels of ZNF268 expression (75% with ++/+++ staining) compared with the expression levels observed in the normal tissues (75% with −/+ staining; Fig. 1A and B). These results suggested that ZNF268 was overexpressed in human ovarian cancer tissues.

### ZNF268-knockdown promotes SKOV-3 cell growth

**Figure 2.**
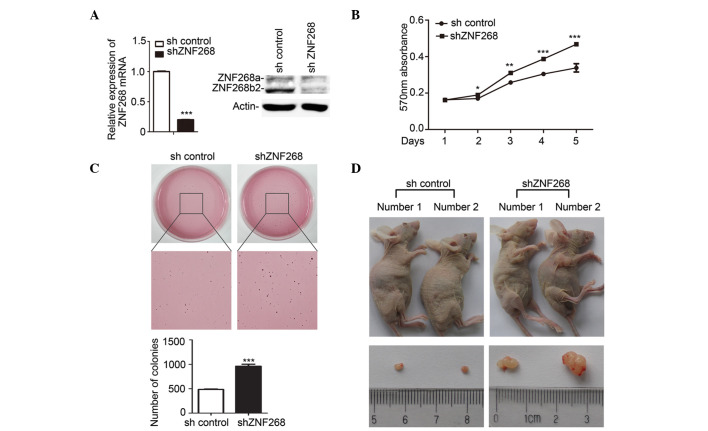
ZNF268-knockdown enhances SKOV-3 cell growth in *in vitro* models and *in vivo* xenografts. (A) ZNF268-knockdown was confirmed at the mRNA and protein levels. SKOV-3 cells were infected with lentiviral particles carrying either the ZNF268 hairpin (shZNF268) or control (sh control) sequences and subjected to real-time PCR (left panel) and a western blot (right panel) analysis to observe the ZNF268-knockdown. An anti-SD antibody was used to detect the ZNF268a/ZNF268b2 protein levels. (B) ZNF268-knockdown increased SKOV-3 cell viability. SKOV-3 cells were seeded at 3.0×10^3^ cells per well in 96-well plates. The growth rate was determined using a 3-(4,5-dimethylthiazol-2-yl)-2,5-diphenyltetrazolium bromide (MTT) assay at the indicated time-points. (C) ZNF268-knockdown increased colony formation in soft agar. The cells were seeded at a density of 4.0×10^3^ per 60-mm plate. Three weeks later, the cell colonies were stained with 2-(p-iodophenyl)-3-(p-nitrophenyl)-5-phenyl tetrazolium chloride for visualisation (upper panel) and statistical analysis (bottom panel). ^*^P<0.05, ^**^P<0.01, ^***^P<0.001 vs. sh control. (D) shZNF268 SKOV-3 cell xenografts exhibited increased growth. shZNF268 or sh control SKOV-3 cells (5.0×10^6^) were subcutaneously injected into nude mice. After 40 days, the mice were sacrificed and the tumours were removed, as shown in the bottom panel.

To investigate the biological function of ZNF268 overexpression in human ovarian cancer tissues, a ZNF268-knockdown was established in the ovarian cancer SKOV-3 cells (shZNF268) using the siRNA method (Fig. 2A). The expression of the ZNF268 mRNA and the levels of the proteins (ZNF268a and ZNF268b2) encoded by the ZNF268 gene were decreased in the shZNF268 cells (Fig. 2A). Next, the effect of ZNF268-knockdown on SKOV-3 cell growth was analysed using the MTT assay. The results showed that the growth rate of the shZNF268 cells was increased two days subsequent to the cells being plated, and the greatest increase in growth rate occurred subsequent to five days (Fig. 2B). The ability to form colonies in soft agar is considered to be an important characteristic of tumour growth *in vitro*. Therefore, the ability of the shZNF268 SKOV-3 cells to form colonies in soft agar was examined. Consistent with the results of the MTT assay, the number of colonies was significantly increased in the shZNF268 SKOV-3 cells compared with the sh control cells (Fig. 2C).

To further study the function of ZNF268, an *in vivo* xenograft model was used. Following the subcutaneous injection of the SKOV-3 cells into six Balb/c-nu mice per group, the mice in the shZNF268 group developed tumours earlier than the mice of the sh control group (day 12 in the shZNF268 group vs. day 18 in the sh control group). In each group, two mice had developed clear tumours at the time they were sacrificed (Fig. 2D). Consistent with the *in vitro* results, the subcutaneous injection of the shZNF268 SKOV-3 cells into the nude mice resulted in increased tumour growth (Fig. 2D). Together, these results demonstrated that ZNF268-knockdown increases SKOV-3 cell growth in *in vitro* models and *in vivo* xenografts.

### ZNF268-knockdown alters SKOV-3 cell cycle progression

**Figure 3.**
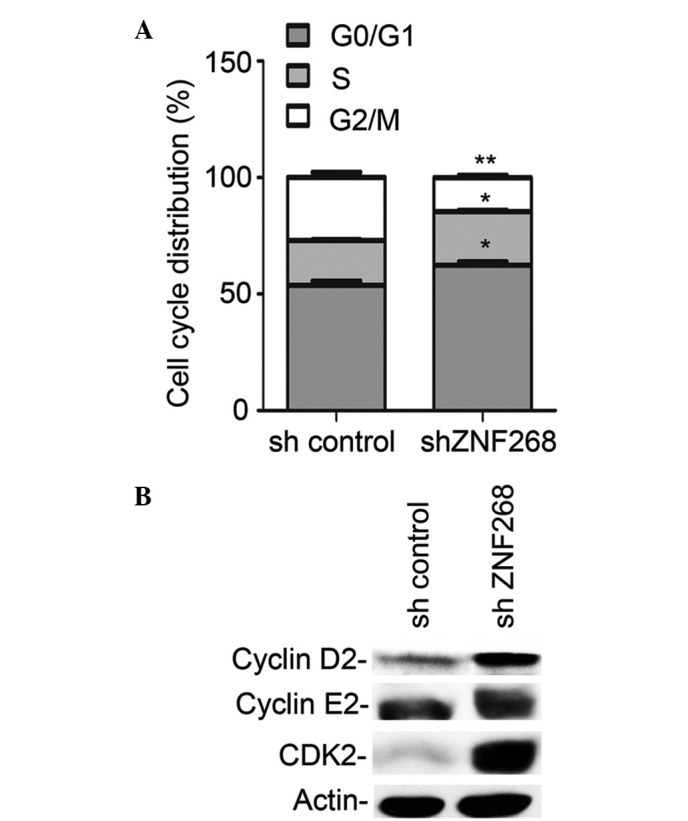
Effects of ZNF268-knockdown on cell cycle progression. (A) Cell cycle distribution of sh control and shZNF268 SKOV-3 cells. The cells were collected during the exponential growth phase, stained with propidium iodide (PI) and analysed by flow cytometry. The histograms represent the percentage of total cells in each phase of the cell cycle. ^*^P<0.05, ^**^P<0.01 vs. sh control. (B) Effects of ZNF268-knockdown on the proteins regulating the cell cycle. Whole cell lysates were prepared and subjected to western blot analysis with the indicated antibodies.

To further determine the potential mechanism by which ZNF268-knockdown increased cell growth, the effects of ZNF268-knockdown on cell cycle progression were analysed. The cells were stained with PI and analysed using flow cytometry. Among the shZNF268 cells, the proportion of cells in the G_0_/G_1_ and S phases of the cell cycle increased as the proportion of the cells in the G_2_/M phase decreased (Fig. 3A). Consistent with these observations, increases in the expression of positive regulators of cell cycle progression, including cyclin D2 and CDK2 (which were significantly increased) and cyclin E2 (which was marginally increased) were also observed (21–23) in the shZNF268 cells (Fig. 3B). These results indicated that ZNF268-knockdown may increase SKOV-3 cell growth by promoting cell cycle progression.

### ZNF268-knockdown suppresses SKOV-3 cell migration in vitro

**Figure 4.**
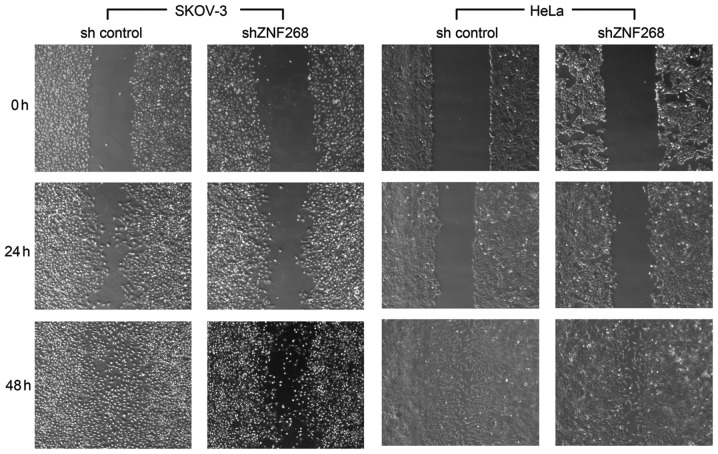
ZNF268-knockdown inhibits SKOV-3 cell migration. Representative images of scratch wound assays performed in SKOV-3 cells (left panel) and HeLa cells (right panel) were obtained at 100× magnification at the indicated times.

The scratch wound assay is a simple and reproducible method for measuring cell migration (24). The function of ZNF268 was investigated in SKOV-3 cell migration using the scratch wound assay. As shown in Fig. 4, migration was decreased in the shZNF268 SKOV-3 cells at 24 h and 48 h post-scratch, suggesting that ZNF268-knockdown suppressed SKOV-3 cell migration *in vitro*. ZNF268-knockdown in the HeLa cells was also established in our previous study (8). However, no clear changes in cell migration were observed in the shZNF268 HeLa cells (Fig. 4).

## Discussion

Although considerable progress has been made in cancer research, the mortality rate of ovarian cancer has not improved over the past several decades (15), demonstrating the urgent requirement for an improved understanding of the mechanisms underlying the development of ovarian cancer. The present study revealed several significant roles for ZNF268 in human ovarian cancer development and progression. First, it was observed that ZNF268 is overexpressed in ovarian carcinomas. Second, ZNF268-knockdown was demonstrated to affect biological functions, including the proliferation and migration of ovarian cancer SKOV-3 cells. The results indicate that ZNF268-knockdown may increase the growth rate of SKOV-3 cells by promoting cell cycle progression, suggesting that ZNF268 may inhibit ovarian cancer cell growth in humans. It is well known that oncogenes are usually mutated or expressed at high levels in tumour cells, while suppressor genes are usually inactive (25). Therefore, the inhibitory function of ZNF268 on cell proliferation may appear controversial due to its high expression levels in ovarian carcinomas. However, exceptions to this rule exist in which there is no direct correlation between function and expression. For example, the tumour suppressor gene maspin is not detected in normal human pancreatic cells, but is highly expressed in pancreatic cancers (26). The tumour suppressor PTEN also shows positive expression in ∼80% of squamous cell cervical carcinomas (27,28) and is inactivated by the negative regulator SIPL1 during cervical tumourigenesis (29). Other negative regulators may interact with and inhibit the activity of ZNF268 in the same way that SILP1 interacts with and inhibits PTEN. Using an immunohistochemistry assay with anti-SD that recognizes the ZNF268a and ZNF268b2 isoforms (6) the present results demonstrated that total ZNF268 protein was overexpressed in ovarian cancer and that ZNF268 (ZNF268a/ZNF268b2)-knockdown increases the growth of SKOV-3 cells. Our previous study showed that the two isoforms (ZNF268a/ZNF268b2) have varying expression patterns and function differently in human cervical cancer development (8). Further experiments should be performed to elucidate the functions of these two isoforms in ovarian carcinogenesis, which are likely to aid in the understanding of the mechanism of ZNF268 in SKOV-3 cell proliferation and ovarian carcinogenesis.

The high expression levels of ZNF268 in human ovarian carcinomas may be associated with the ability of ZNF268 to promote cell migration. Due to the asymptomatic nature of the early stages of ovarian cancer and the lack of a reliable method for early detection, the majority of ovarian cancer patients are diagnosed with metastatic disease, the cure rate of which is significantly less than that of non-metastatic disease (17,18). From this perspective, ZNF268-knockdown suppresses SKOV-3 migration, indicating that ZNF268 may serve as a potential therapeutic target for ovarian cancer. However, the function of ZNF268 requires further validation *in vivo* and the molecular mechanism underlying the suppression of migration requires investigation.

Several lines of evidence, based on gene structure, suggest that ZNF268 may be important in human development. First, ZNF268 contains a typical KRAB domain, which is only present in tetrapod vertebrate genomes (2). Second, ZNF268 consists of up to 24 zinc fingers, and the number of zinc finger repeats tends to increase during the evolutionary process (30). Third, no ZNF268 ortholog has been detected in the mouse genome. In the present study, the knockdown of ZNF268 increased SKOV-3 cell growth. This is similar to the effects of ZNF268-knockdown on growth promotion observed in erythroleukemia K562 cells (14), but contrary to the effects of ZNF268-knockdown on the growth of cervical HeLa cells (8). The underlying reason for these differences may be that the functional mechanism varies in these cells; ZNF268-knockdown increases the proportion of cells in the S phase in SKOV-3 and K562 cells (14), but inhibits HeLa cell cycle progression by causing G_0_/G_1_ phase arrest (8). Notably, the proportions of the SKOV-3 cells in the G_0_/G_1_ and G2/M phases were also increased and decreased, respectively, by ZNF268-knockdown, suggesting that ZNF268-knockdown also affects the other phases, and their coordinative effect on cell cycle progression may explain the increased SKOV-3 cell growth. In addition, the role of ZNF268 in cell migration has also been demonstrated to be different in the SKOV-3 and HeLa cells. These results suggest that ZNF268 may act as a multifunctional molecule in humans by utilising various mechanisms in different cell types.
